# The Traumatic Abdomen

**Published:** 1919-01

**Authors:** 


***The Traumatic Abdomen.*** By JOHN B. DEAVER. *Annals of Surgery*, September 1918.

Prostration, pallor, anxious facies, thirst, cold, exquisite pain on moving the body, limited abdominal movement on respiration, rapid pulse of low tension, rigidity, dulness in the flanks, and vomiting are symptoms that leave little doubt of some visceral lesion, but often the picture is not clear in spite of the presence of serious intra-abdominal injury.

Operation is now considered the only chance of recovery in the majority of cases. The dictum seems to be: operate, operate early, operate as near to the front as possible, and make the operation as complete as possible. Shock does not necessarily contra-indicate operation.

In the diagnosis of a suspected abdominal lesion, pain is of little aid, and there is apparently no direct relationship between its intensity and the extent of the injury. Pulse and the degree of abdominal rigidity are of importance. The absence of rigidity is now generally recognized as an unfavorable prognostic sign, since it is usually associated with extensive lacerating lesion of the small and sometimes the large intestines.

The site of the wound is not an unfailing indication as to the involvement or otherwise of the abdominal cavity. A foreign body may enter almost any region of the body and traverse and lodge in the abdomen.

A valuable diagnostic point is the consideration of the entrance and the exit wounds, where both are present, and the course and direction of the track; that is to say, the plane of abdominal involvement and the structures that may have been traversed.

The removal of clothing, emptying of the bladder, and washing the skin, preferably with a carbolic lotion or iodine, are about all that can be done in the majority of instances. Morphia and atropine are given hypodermically, and if possible one hour before the anesthetic, preferably ether, is administered. Satisfactory results have been obtained with intravenous injections of $NaHCO_3$ before anesthesia.

As a rule, a median or paramedian longitudinal incision is selected, and should be ample so as to allow free access to the cavity, which in turn means rapid work.

It should be remembered that the peritoneum does its best work when unhampered, which means limited drainage, if any. Lavage of the abdominal cavity is not generally advocated.

As to the involvement of one or the other viscus, it appears that in war injuries of the abdomen the small intestine is the most frequently injured. Suture is the proper method of treating these wounds; resection being reserved for cases with numerous perforations close together.

The large intestine when wounded usually presents only a single tear or perforation rather than a complete section of the gut.

Colon wounds are characterized by their tendency to localization. Colotomy at the site of injury is required when the wound is extensive; otherwise, suture reinforced by omental graft seems to be the chosen method.

It seems that suture is the preferred method of dealing with perforating gunshot wounds of the stomach.

Wounds of the rectum, when extraperitoneal, are treated in the usual manner by establishing drainage after the wound has been opened up; and when intraperitoneal, by suture followed in certain cases by colotomy.

Bladder wounds are fortunately very rare, for their mortality is very high. Extraperitoneal injury, indicated usually by hemorrhage into the bladder, may be treated by catheterization or by perineal section; intraperitoneal injury, however, demands immediate operation. Wounds of the spleen usually require splenectomy.

Post-operative treatment of the traumatic abdomen does not essentially differ from the regimen in use for other abdominal operations. The great advantage gained from war experiences is the removal of any hesitancy in exploring the abdomen, and with it the reduction of about 10 per cent in the mortality in this type of injury.

